# Inter-agency perspective: Translating advances in biomarker discovery and medical countermeasures development between terrestrial and space radiation environments

**DOI:** 10.1016/j.lssr.2022.06.004

**Published:** 2022-06-14

**Authors:** Andrea L. DiCarlo, Lisa S. Carnell, Carmen I. Rios, Pataje G. Prasanna

**Affiliations:** a Radiation and Nuclear Countermeasures Program (RNCP), National Institute of Allergy and Infectious Diseases (NIAID), National Institutes of Health (NIH), 5601 Fishers Lane, Rockville, MD, 20852 United States of America; b Biological and Physical Sciences Division, National Aeronautics and Space Administration (NASA), 300 E Street SW, Washington, DC, 20546 United States of America; c Radiation Research Program (RRP), National Cancer Institute (NCI), National Institutes of Health (NIH), 9609 Medical Center Drive, Bethesda, MD, 20892 United States of America

**Keywords:** Space, Radiation, Medical countermeasures, Chronic, Cancer, Biomarkers

## Abstract

Over the past 20+ years, the U.S. Government has made significant strides in establishing research funding and initiating a portfolio consisting of subject matter experts on radiation-induced biological effects in normal tissues. Research supported by the National Cancer Institute (NCI) provided much of the early findings on identifying cellular pathways involved in radiation injuries, due to the need to push the boundaries to kill tumor cells while minimizing damage to intervening normal tissues. By protecting normal tissue surrounding the tumors, physicians can deliver a higher radiation dose to tumors and reduce adverse effects related to the treatment. Initially relying on this critical NCI research, the National Institute of Allergy and Infectious Diseases (NIAID), first tasked with developing radiation medical countermeasures in 2004, has provided bridge funding to move basic research toward advanced development and translation. The goal of the NIAID program is to fund approaches that can one day be employed to protect civilian populations during a radiological or nuclear incident. In addition, with the reality of long-term space flights and the possibility of radiation exposures to both acute, high-intensity, and chronic lower-dose levels, the National Aeronautics and Space Administration (NASA) has identified requirements to discover and develop radioprotectors and mitigators to protect their astronauts during space missions. In sustained partnership with sister agencies, these three organizations must continue to leverage funding and findings in their overlapping research areas to accelerate biomarker identification and product development to help safeguard these different and yet undeniably similar human populations – cancer patients, public citizens, and astronauts.

## 1. Introduction

Given the importance of ensuring the protection of humans from radiation injury, strong collaborations have been forged between U.S. Government funding agencies with mandates to develop products to protect, mitigate, and/or treat radiation-induced normal tissue injuries. There has been a strong track record of interactions between the National Aeronautics and Space Administration (NASA) and the National Institutes of Health (NIH), including the National Cancer Institute (NCI) and the National Institute of Allergy and Infectious Diseases (NIAID) - organizations that have been tasked with understanding mechanisms of radiation-induced biological damage and exploring pathways for the development of drugs to address radiation injuries. Past synergies have included co-sponsoring scientific meetings, co-funding research, and involvement through inter-agency grant/contract review teams and working groups. Several publications have resulted from these prior cooperative efforts (Carnell et al., 2017; Yoo et al., 2014). In 2007, a memorandum of understanding was established between NIH and NASA^1^ to formalize interactions with each organization establishing areas of cooperation, including the “development of biomedical research approaches and clinical technologies for use on Earth and in space.” NASA and the U.S. Department of Health and Human Services (HHS) also signed an interagency agreement in 2018 to share innovative ideas for addressing science and technology challenges and to coordinate and collaborate on research that could improve human health on Earth and in space.^2^ Finally, since 2017 the NIAID/NASA Medical Countermeasures Working Group, with representation from NCI and other parts of HHS, has endeavored to rapidly and continually share information concerning research advances, to accelerate activities for all agencies.

Although the core mission of these organizations is similar, each directs research efforts toward the specific needs of its target human population. For example, NCI’s interest in radiation stems from the use of localized, high-dose, gamma, x-ray, or proton radiotherapy in cancer treatments. Since incidental damage to surrounding normal tissues can limit the amount of radiation therapy delivered to a tumor, developing approaches using short-term administration of radioprotectants that are not tumor-protective is essential (Prasanna et al., 2012). Additionally, since the time of radiation exposure is known during radiotherapy, it is possible to administer radioprotectors before irradiation.

Similarly, NASA’s concern is the protection of astronauts who will be exposed to low dose but extended periods of irradiation with high linear energy transfer (LET) radiation. The primary risks of concern include carcinogenesis, central nervous system (CNS) effects resulting in potential in-mission cognitive or behavioral impairment and/or late neurological disorders, degenerative tissue effects including circulatory and heart disease, as well as possible immune system decrements impacting multiple aspects of crew health. These outcomes also represent concerns for late effects of irradiation similar following a course of radiotherapy or after unanticipated low-dose exposures during a radiation incident. Characterization and mitigation of these risks require a significant reduction in the large biological uncertainties of chronic (low-dose rate) high charge and energy (HZE) exposures and validation of medical countermeasures (MCMs) in a relevant space environment (Simonsen et al., 2020). Although exposure levels are much lower than those used in radiotherapy, the duration of exposure is far longer. The intent of using mitigators is to prevent and/or reduce late effects thought to be associated with chronic, low doses of radiation. Thus, it is necessary to have products that are safe when dosed continuously because the time during a spaceflight when a harmful exposure may occur can be difficult to predict.

Finally, the mission of the Radiation and Nuclear Countermeasures Program (RNCP) within the NIAID is to address radiation injuries following prompt, high-dose exposure of civilian populations during a radiological or nuclear incident. Because it is impossible to predict when these exposures will occur, use of prophylactic (pre-exposure products is not feasible; therefore, drugs that can mitigate (given post-irradiation but before symptoms manifest) or treat (given after clinically detectable injury) are of greatest interest. Like the NCI, high doses of radiation are of concern; however, the exposures are anticipated to be nearly total-body. NIAID’s primary focus is on developing effective approaches to improve survival and reduce the risk of major morbidities.

Clearly, given limited budgets available for radiation research, to maximize opportunities for synergy, it is incumbent on NASA, NCI, and NIAID to leverage investments across mission spaces when possible. In addition, with a limited number of radiation researchers, principal investigators are often involved in, and receive funding from, all three agencies to carry out research projects that have some overlap. To that end, the authors have attempted to identify radioprotectors, radiomitigators, and radiotherapies in their respective portfolios, to address NASA’s concerns in the care of astronauts exposed to radiation in long-duration space travel. It should be noted that the focus of the present work is on drugs with potential use to mitigate damage resulting from chronic, long-term exposures. Therefore, a discussion of approaches to minimize acute radiation syndrome (ARS) risks to astronauts from solar particle radiation is not considered in depth. A separate review addressing this area has been released by the NASA Human Research Program (National Aeronautics and Space Administration 2016). Furthermore, gaps in each mission space are identified herein, and the need for shared approaches across these organizations to address them.

## 2. Background

**Fig. 1.**
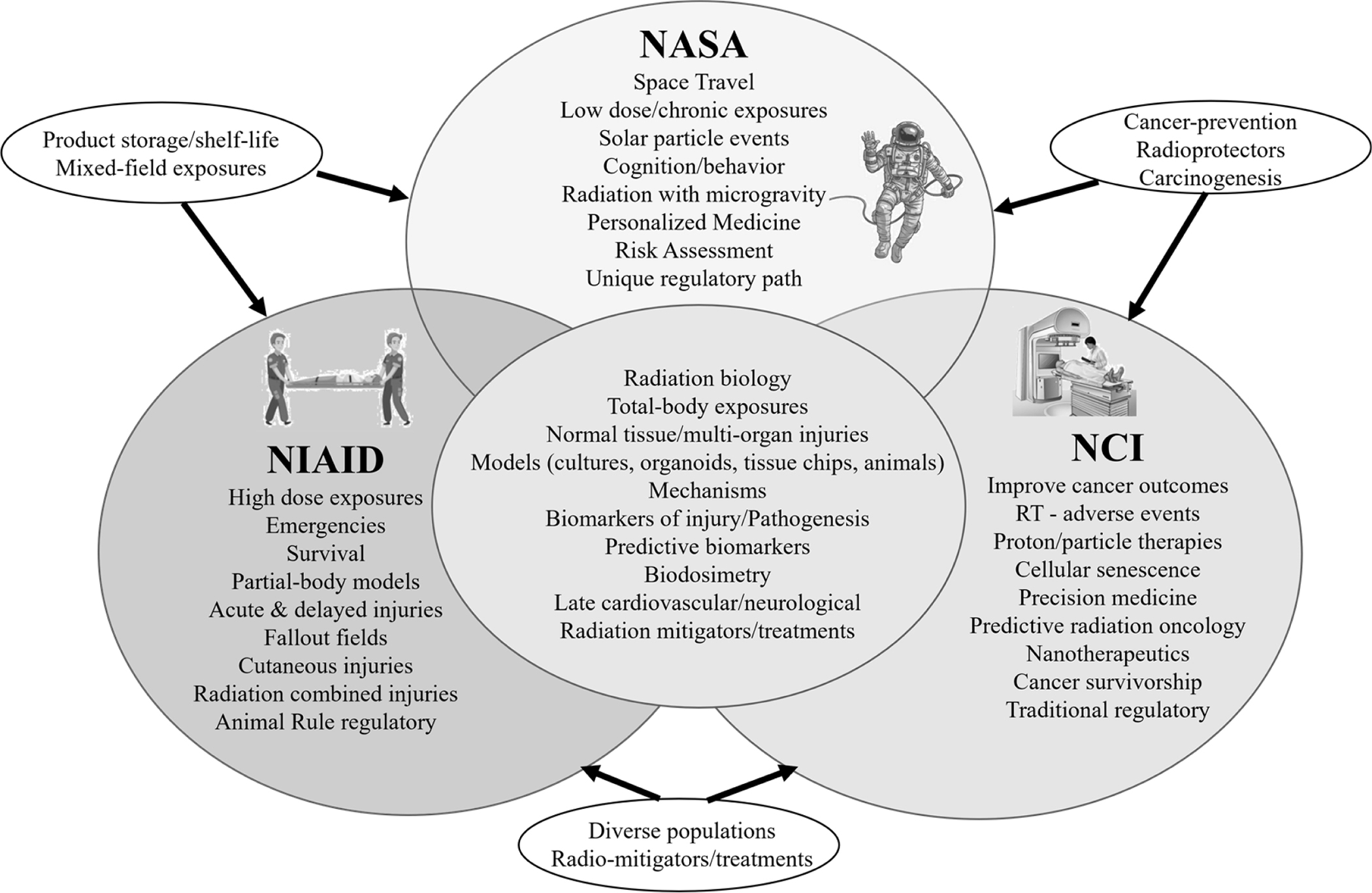
Areas of overlap between the missions of NASA, NIAID, and NCI.

The importance of MCM development and biomarker identification for both acute and long-term effects of radiation align with the missions of NIAID, NASA, and NCI (Fig. 1). NASA is concerned with acute exposures from solar particle events (SPEs), and delayed effects from these exposures could impact the quality of life for the crew upon returning to Earth. NASA is also interested in the bodily effects of prolonged exposure to space radiation during long missions. At the same time, NCI is concerned with roles of radiation in cancer prevention, detection, diagnosis, treatment, and survivorship. Charged with treating civilian casualties after a radiological or nuclear incident, NIAID’s efforts are focused primarily on the acute and delayed effects of high dose-irradiations to large portions of the body.

### 2.1. NASA

NASA has identified five hazards to astronauts associated with long-duration, deep space exploration missions (radiation, isolation and confinement, distance from Earth, Gravity (or lack thereof), and hostile/closed environments). Three key radiation-associated risks have also been outlined: 1) carcinogenesis; 2) cardiovascular disease and other degenerative tissue effects; and 3) acute (in-flight) and late central nervous system effects (e.g., changes in cognition, motor function, behavior, or neurological disorders). These risks also align well with those of NIAID and NCI. The primary challenges for NASA are to understand the severity of these risks in a space-relevant environment, evaluate MCMs that may provide radioprotection or mitigation, and identify biomarkers to indicate if/when an MCM may need to be administered during a mission. It is also imperative to determine any proposed MCM’s dosage, delivery, storage, and shelf-life since many space missions will have limited payload and refrigeration capacity. Therefore, selecting critical-need pharmaceuticals and identifying biomarkers to determine the timing of intervention and avoid treatment-related toxicities will be paramount. Continued interactions with NIH programs exploring therapeutics for normal tissue injuries will provide new approaches to be considered for astronaut health in a radiation environment.

### 2.2. NIAID

The mission of NIAID’s RNCP is to support early to mid-stage research to develop MCMs and biodosimetry tools for U.S. Food and Drug Administration (FDA) approval, with an emphasis on products to treat or mitigate radiation injuries when administered post-irradiation, radionuclide decorporation agents, and biodosimetry tools and biomarker discovery for triage and to predict biological impacts of exposure. Several of these topics represent areas of overlap with NCI and NASA (e.g., minimization of normal tissue injuries, and biomarker identification). Another area of focus for NIAID, and a shared interest of NASA and NCI are the delayed effects of acute radiation exposure, including lung complications. NIAID has funded intramural studies at NCI on long-term health outcomes resulting from chronic, lower dose irradiation, since people could spend significant time in a lower dose, fallout environment after a nuclear incident (Simon et al., 2022; Beck et al., 2022; Land et al., 2015; Drozdovitch et al., 2015). These findings can be used to anticipate potential outcomes following chronic exposures of NASA space flight personnel, and possible late effects in radiotherapy patients. In addition, the strong partnership between NIAID and NASA staff has helped both agencies to keep abreast of significant developments in each program, including exciting research findings and shared policy decisions.

### 2.3. NCI

More than 50% of cancer patients are treated with radiotherapy, often combined with chemotherapy, immunotherapy, and/or surgery. Therefore, NCI’s Radiation Research Program (RRP) was established as a nexus for funding extramural research activities related to radiation therapy. Although dose prescriptions to a tumor or tissue are generally based on a population average, radiation therapy is now pursuing precision medicine and predictive oncology, where doses are individualized or stratified to a cohort (Coleman et al., 2018). Since many patients suffer from adverse treatment effects, which could be acute, intermediate, or late (Prasanna et al., 2012), the RRP has supported predictive biomarker development (Prasanna et al., 2020; Prasanna et al., 2014), along with radioprotectors and mitigators to reduce treatment-related toxicities and improve patient outcomes (Prasanna et al., 2015; Coleman et al., 2021). Cancer survivorship will expand with improvements in treatment, with one estimate suggesting >26 million cancer survivors by 2040 (Bluethmann et al., 2016). Establishment of tracking in these populations will provide an opportunity to study late radiation impacts, including second cancers (Singh et al., 2017) and cardiovascular disease (Spetz et al., 2018). Exploring outcomes in these patient populations can inform long-term impacts of continuous, lower doses of radiation exposure in individuals involved in NASA long space flights and provide insight into possible late effects in populations unexpectedly exposed to radiation during a public health emergency.

## 3. Cross-cutting models and tools

### 3.1. Preclinical models

A shared challenge in radiation research among agencies is replacing, reducing, and responsibly conducting animal research while identifying, and developing unproven therapies for human use. To address this requirement, many models have been established to look at terrestrial and space-based radiation, ranging from in vitro cell cultures, tissue chips, and invertebrates, to small (rodents) and large (nonhuman primates, dogs, minipigs, etc.) laboratory animals to humans. In addition, infrastructure for studying the effects of high-LET radiation exposures is limited and expensive, and thus a challenge when developing large animal models, so leveraging studies on these species in other radiation scenarios (such as radiotherapy in the clinic or unanticipated exposure during a radiological or nuclear incident) is essential. In many instances, researchers receive concurrent support from NASA, NCI, and NIAID, and can use the animal models they have developed to contribute meaningful data for all three agencies.

The 2021 NASA trans-agency research announcement, “Extended Longevity of 3D Tissues and Microphysiological Systems (MPS) for Modeling Acute and Chronic Exposures to Stressors,^3^ represents an effort sponsored by NASA’s Space Biology Program, with the participation of NASA’s Human Research Program. Complex human in vitro models such as tissue chips or microphysiological systems (MPS) are three-dimensional (3D) systems constructed of cells grown on bioengineered platforms that mimic in vivo tissue architecture and physiological conditions. These platforms can incorporate complex in vivo factors, including extracellular scaffolding, 3D structures, cellular interactions, perfusion, biomechanical stresses, electrical stimulation, and hormone/inflammatory responses. The purpose of the announcement was to solicit research supporting common cross-organizational goals by focusing on adapting existing MPS to extend their longevity to at least six months, while thoroughly testing and validating the models for acute and chronic stressors. Contract awards reflect collaboration across multiple agencies including NCI and NIAID. Each participating organization has unique and specific applications for tissue-engineered models, yet they all identified common interests resulting in this multi-organizational effort, and the resulting research findings are expected to benefit all the mission spaces.

### 3.2. Biomarkers of injury

Biomarkers in biomedical research are integral to understanding disease pathogenesis and healing (Robb et al., 2016). Once biomarkers are identified, evaluated, and qualified, they can help bridge preclinical data across species and predict human clinical outcomes. As defined by the NIH-FDA Biomarker Working Group in the joint document, BEST (**B**iomarkers, **E**ndpoint**S**, and other **T**ools) Resource,^4^ biomarkers are indicators of normal biological and pathogenic processes or responses to an exposure or intervention, including therapeutics (Robb et al., 2016). Biomarkers can be molecular, histologic, radiographic, or physiologic characteristics and do not necessarily assess how a patient feels, functions, or survives (Califf, 2018). Molecular patterns, gene expression profiles, and miRNA signatures have been explored for possible use in radiation dose reconstruction, disease prognosis, prediction of radiation sensitivity, and use in medical triage. In addition, study of biomarkers to determine if and when MCMs should be administered during a radiation emergency also inform on exposures during a spaceflight mission, and may reduce the need to take prophylactic MCMs and allow appropriate medical intervention. Biomarkers could also help determine individual sensitivity from radiation exposure and guide both determination of fitness for radiation exposure (e.g., during therapy or space flight), and precise delivery of MCMs for each victim, radiotherapy patient or space crew member. To this end, NASA, NCI and NIAID have worked together to explore different models for radiation-induced injuries, such as hematopoietic (DiCarlo et al., 2019), cutaneous (DiCarlo et al., 2020), lung (Cassatt et al., 2022), and late carcinogenic endpoints (Yoo et al., 2014).

### 3.3. Data mining/analytics

Discovery and identification of areas of interest for generating leads to develop therapies will need to be accomplished by employing data analytics across numerous data sets. NASA’s GeneLab repository hosts space biology and related laboratory datasets and is utilized by multiple agencies worldwide. This interactive, open-access database allows scientists to upload, download, store, search, share, transfer, and analyze “omics” data from spaceflight and corresponding analog experiments. Although a vital shared resource, NASA does not have the volume of human flight data necessary to achieve statistical significance for many inquiries. Working with national and international communities to combine data in a central database will help develop new approaches. Fortunately, there is potential for collaboration with NCI, and NIAID in this area. The NCI Cancer Research Data Commons^5^ provides access to genomic, proteomic, comparative oncology, imaging, etc., in addition to data from NCI programs such as The Cancer Genome Atlas.^6^ These projects address several NCI challenges, including understanding molecular bases of crucial protein interactions, developing predictive models for drug response, and automating analysis and extraction of information from patient records to determine optimal treatment strategies.^7^ NCI staff are discussing with NASA program representatives ways to explore these databases for relevant information on radiation exposures of interest to both agencies.

NIAID has developed the Immunology Database and Analysis Portal (ImmPort)^8^ resource, which provides experimental data, metadata, and analysis tools on basic and clinical immunology research that includes radiation information. ImmPort is open to the public for sharing research data and creates an integrated environment that broadens the use of scientific data and advances hypothesis-driven/generating research. There is currently an effort underway to connect ImmPort with NASA GeneLab and NCI repositories described above to provide researchers curated access to volumes of data that can be mined for future radiation studies.

## 4. Knowledge gained from epidemiology studies addresses space risk assessment

Radiation epidemiological health risk assessments conducted by the U.S. Government have included studies of Japanese A-bomb survivors and humans exposed during nuclear emergencies, occupational exposure cohorts, astronauts, populations living in high background radiation areas, and nuclear medicine and radiotherapy patients (Ali et al., 2020). For example, cancer risks in the A-bomb lifespan study cohort continue to rise throughout life (Ozasa et al., 2012); however, clearly defining carcinogenic risks from low-dose irradiation has been challenging A literature review published by NCI researchers within the Division of Cancer Epidemiology and Genetics considered studies with mean radiation doses less than 100 mGy and found an excess relative risk of greater than 1.0 for most solid cancers and leukemia (Berrington de Gonzalez et al., 2020). This is consistent with the recent million-person study of U.S. radiation workers and atomic veterans that suggests leukemia and lung cancer risks are lower from chronic than acute exposures, and that low-level radiation exposures are associated with circulatory diseases (Little et al., 2012). NIAID has supported epidemiology work carried out by NCI researchers on health effects in Marshall Islanders exposed to the fallout following the Castle Bravo nuclear test (Simon et al., 2022), individuals exposed during Nevada Atomic Testing (Beck et al., 2022), and the Semipalatinsk Nuclear Test Site (Land et al., 2015), and exposed populations living near Chernobyl (Drozdovitch et al., 2015). These studies have contributed to a better understanding of how persistent, low-dose exposures can be harmful. Because it is difficult to assess the long-term impact of space radiation on flight personnel, these studies assessing late effects resulting from exposure to other forms of radiation provide insight into what could be experienced by astronauts after returning from long duration space missions. The NASA Space Radiation Laboratory (NSRL) at Brookhaven National Laboratory has been expanded through development of a galactic cosmic rays (GCR) facility designed to provide a mixed spectrum of ion beams to simulate the space radiation environment (Simonsen et al., 2020). NSRL provides researchers the ability to conduct high-LET, mixed field radiation studies on the ground, to better understand space radiation effects on biology. understand space radiation effects and perform long-terms animal studies under controlled conditions. While NSRL can provide access to high-LET mixed field exposures, the dose-rate expected in space is significantly lower than the facility can provide, hence, the ideal exposure scenario will be conducted on future Artemis missions designed to take astronauts to the lunar surface, Gateway, and beyond.

## 5. Repurposing licensed products

Inherent to NASA’s challenges in developing MCMs for use in space is the need to minimize the mass of carried products. For this reason, it is of great interest for potential radiation MCMs to have multiple space crew uses. The ideal drug, for example, will provide cross-protection through antioxidants that provide free radical scavenging, common pathways (e.g., inflammatory), or other biological pathways that intersects multiple risks. If an MCM were able to be used as a radioprotectant in addition to another health indication, that kind of repurposing would be desirable. Both NIH and NASA have prioritized repurposing of products for other clinical indications so that a large body of safety and efficacy in humans physician familiarity can be leveraged. In the case of NASA, a product’s approval status can inform on its safe extended use in the space environment and accelerate the timeline for transition to operations. A 2017 meeting held by NIAID on this topic outlined several already licensed drugs that could be considered as radiation treatment approaches (DiCarlo et al., 2018). In addition, a 2017 review of the potential for the use of common drugs to protect during spaceflight has reviewed in more detail several of the approaches discussed below (McLaughlin et al., 2017).

## 6. Overlapping mechanisms of radiation injury drug classes with space mission utility

In leveraging investments by the NIH in approaches of potential interest to NASA, it is essential to note that such treatments may need to be administered over time frames of months to years to ensure that protection was consistent throughout, for example, an extended space flight mission to Mars. For that reason, products that are known to be safe with extended dosing provide the most appropriate selections for further study. Many approaches with these known long-term safety profiles fall into the general category of nutraceuticals. However, several products in constant and daily use by individuals worldwide also fall into this category (e.g., antibiotics, analgesics, anti-hypertensives). Although several products detailed below were initially tested in a high-dose, terrestrial irradiation setting, some have also shown promise for more chronic, lower-dose, and dose-rate irradiation exposures, and have demonstrated benefits in a space-irradiation context. These selected approaches that have received NASA and/or prior NIH funding are discussed below and outlined in Table 1.

### 6.1. Anti-oxidants

Under physiological conditions, low reactive oxygen species (ROS) levels are produced due to normal cellular processes. ROS existing as free oxygen or nitrogen oxide radicals primarily function as signaling molecules that induce cell differentiation and apoptosis, among other activities. However, exposure to stressors such as radiation can trigger an imbalance in free radical generation and their removal, leading to DNA damage (Jakubczyk et al., 2020), with the potential to induce early and late effects (Hoorelbeke et al., 2020). It has been established that radiation exposure in all its forms leads to an immediate and chronic state of oxidative stress. The interaction of radiation with water molecules within the body leads to ROS generation, which, although short-lived, can wreak cellular havoc (Simone et al., 2009). Similarly, a chronic state of radiation-induced oxidative stress (Riley, 1994) can lead to altered homeostasis, which can continue for many hours to months beyond initial exposure (Azzam et al., 2012). This oxidant injury is non-specific, with all intracellular elements (e.g., proteins, lipids, carbohydrates) subject to the damage.

For this reason, antioxidant approaches have been investigated to modulate radiation-induced intracellular perturbations. Several popular candidates include nutraceuticals (e.g., vitamins (Changizi et al., 2019; Sihver and Mortazavi, 2021; Singh et al., 2013), melatonin (Kumar et al., 2022), flavonoids (Landauer et al., 2019), and other dietary approaches (e.g., curcumin (Amini et al., 2018; Jelveh et al., 2013), and flaxseed (Christofidou-Solomidou et al., 2011). This predominantly “generally regarded as safe” (GRAS) class of products has volumes of safety data and known low toxicity. Such products may be amenable to NASA since they could be easily included in the essential diet of a space traveler.

#### 6.1.1. Nutraceuticals/dietary modifications

Several vitamin approaches mentioned above are covered in detail in another paper in this issue (Singh et al.). Dietary supplementation with Bowman-Birk protease inhibitors derived from soybeans (Shaghagi et al., 2021; Kennedy, 2021), L-selenomethionine, or a combination of selected antioxidant agents (Guan et al., 2004; Kennedy et al., 2007) has shown promise to prevent the decrease in antioxidants in the plasma of mice exposed to proton or HZE particle radiation. Adding nutritional foods, such as blueberries, to crew diets may provide added benefits to help reduce ROS and mitigate radiation-induced CNS effects, as reported in animal studies using space-relevant radiation exposures (Poulose et al., 2017). In one study, feeding dried plums to mice mitigated radiation-induced loss of bone and demonstrated potential as a radiomitigator for protons and HZE particle radiation (Schreurs et al., 2016). Flaxseed is bioactive lignin that is both an antioxidant/anti-inflammatory signaling agent (Turowski et al., 2015). It has been in clinical trials for non-small cell lung cancer to protect normal tissue (NCT00955942) and manage cardiovascular risk and hypotension (Berman et al., 2013). NASA recently funded a Phase II SBIR study to investigate the potential of LGM-2605, synthetic flaxseed, as a potential prophylactic radioprotector from the mixed field and GCR effects (described in greater detail below) (Chatterjee et al., 2019).

#### 6.1.2. LGM2605

LGM2605 (LignaMed, Philadelphia, PA) is a synthetic form of a natural component typically found in dietary flaxseed; thus, its excellent safety profile is well-established. Initially studied in cell-free (Mishra et al., 2014) and in vitro models of radiation injuries (Chatterjee et al., 2019), it became apparent that the radiation protection offered by the product (and its dietary-derived precursors) was significant. Later in vivo work focused on mitigation of radiation-induced lung injuries (Lee et al., 2009; Christofidou-Solomidou et al., 2011), and an *ex vivo* organ culture model of human lungs showed protection against proton irradiation (Velalopoulou et al., 2017). Of importance is the finding that the drug could reduce tissue injuries from exposure to radiation with high relevance to NASA, where it demonstrated the ability to blunt high-LET radiation-induced activation of the inflammasome in a lung vascular model (Chatterjee et al., 2019). Therefore, this product seems to be a reasonable option for NASA’s consideration.

#### 6.1.3. Enterade

The University of Florida, which first created the Gatorade sports drink, also recently developed another nutritional plant-based drink for cancer patients, Enterade. It is a commercially available amino acid-based oral rehydration fluid that has been shown to alleviate GI complications experienced by patients undergoing chemo- and/or radiation therapy (De Filipp et al., 2021). Preclinical studies have also shown that Enterade can reverse radiation-induced injury that leads to a breakdown in GI structure (Gupta et al., 2020), increase weight gain and provide a survival benefit in mice (Yin et al., 2014). It has also been studied with funding from the National Space Biomedical Research Institute in experiments that found that the drink given as a daily gavage improved electrolyte balance and GI absorption of nutrients in irradiated animals (Yin et al., 2016).

#### 6.1.4. Ketones

A method for reducing free radical damage occurs through the metabolism of ketone bodies. Elevated levels of ketone bodies can be pathological in disorders such as diabetes or maybe the result of a diet low in carbohydrates. Ketone ester has been evaluated as a dietary supplement to address oxygen toxicity (Stavitzski et al., 2021) and is considered by the FDA to fall into the GRAS category (Veech et al., 2017). A novel ketone ester product has been tested for efficacy in preclinical studies in mice for hematopoietic ARS. It is hypothesized that ketone esters can protect hematopoietic tissue and thus improve chances of survival in the animals by quenching the cascade of free radicals induced by ionizing radiation.^9^ Use of ketones in cancer therapies has also been reviewed (Klement, 2018), where its use has been linked to a lower incidence of normal tissue injuries during radiation therapy.

#### 6.1.5. Superoxide dismutase (SOD) mimetics

SOD mimetics, such as Mn porphyrins, can have diverse reaction potentials in vivo and modulate cellular redox regulatory signaling networks (Batinic-Haberle and ME, 2019). They have been shown to have different effects on cancerous versus normal cells (Batinic-Haberle et al., 2018). One of these compounds, BMX-001, is being developed as a radioprotector to prevent normal tissue damage during cancer treatments. The drug is undergoing a clinical trial for patients with squamous cell head and neck cancer going through radiotherapy with concomitant cisplatin (National Clinical Trial (NCT) 02,990,468) and also as a neuroprotector for patients with gliomas receiving radiotherapy plus temozolomide to protect against cognitive deterioration and improve survival (NCT02655601) (Zakeri et al., 2019).

Similarly, another small molecule, SOD mimetic, GC4419 (Avasopasem manganese), is being developed to prevent radiation-induced toxicities. The product recently completed two clinical trials to mitigate severe oral mucositis in head and neck cancer patients undergoing radiation therapy and cisplatin (NCT01921426, NCT02508389) (Anderson et al., 2018), where it reduced incidence, duration, and severity of oral mucositis without interfering with anticancer efficacy of the radiotherapy. Additional information on the Galera compound can be found in another manuscript in this special issue journal (Beardsley et al., 2022).

BIO300 - a synthetic flavonoid (genistein) nano-formulation of a SOD mimetic - is a promising MCM being developed as a mitigator of hematopoietic acute radiation syndrome (H-ARS) and late lung complications (Singh and Seed, 2020). Initially supported by DoD, the product has been tested in patients undergoing radiation therapy for non-small cell lung cancer (NCT02567799) to mitigate pneumonitis and fibrosis (Prasanna et al., 2015; Day et al., 2008; Citrin et al., 2017; Zakeri et al., 2019). The product is currently being tested in COVID-19 patients with post-hospitalization lung complications (NCT 04,482,595).

Finally, the SOD mimetic AEOL 10,150 received U.S. government funding to inform its use as a mitigator of radiation-induced lung damage. The compound, also a Mn porphyrin, is protective in mouse (Murigi et al., 2015), hamster (Sonis, 2001), and nonhuman primate (NHP) (MacVittie et al., 2017; Garofalo et al., 2014) models of radiation injury, with a proposed mechanism of action that involves reduction of innate immune response (Cui et al., 2021). Key to the potential use of this product in a space flight environment is the lack of toxicity observed in preclinical models despite continuous dosing over several months (MacVittie et al., 2017).

#### 6.1.6. Tempol

Another antioxidant agent that could also double as a dietary supplement is a stable, water-soluble nitroxide free radical (Hahn et al., 1992). Tempol as a radio-protector and mitigator has been studied for radiation oncology indications (Hahn et al., 1994), where it has been shown to have antioxidant activity in the protection of normal tissues and as a mitigator of late radiation-induced carcinogenesis known to arise after lower doses of exposure in a mouse model of injury (Mitchell et al., 2012). It is a viable option for long-term administration, having been delivered to irradiated and unirradiated preclinical models across their entire lifespan with no adverse outcomes.

### 6.2. Anti-inflammatories (non-steroidal anti-inflammatory drugs, NSAIDs)

Along with chronic oxidative stress, chronic inflammation has been identified as a critical pathway in the development of cancer and cardiovascular, neurodegenerative, and autoimmune diseases. For this reason, anti-inflammatories are addressed here as potential therapies of interest to NASA.

#### 6.2.1. Aspirin

Researchers have reported that long-term use of aspirin significantly impacts cancer incidence and mortality (Qiao et al., 2018). Aspirin also reduces the incidence of cardiovascular events by ~20% in the general population and high-risk groups (Mitrugno et al., 2017; Camara Planek et al., 2020). NASA has examined aspirin’s efficacy in reducing radiation-induced cancers, and cardiovascular, immune, and behavioral health effects. For example, in a human gastrointestinal (GI) cancer model, mice exposed to ^28^Si-ions and a diet supplemented with aspirin reported a significant reduction in a potent pro-inflammatory molecule but not a reduction in GI tumorigenesis (Suman et al., 2021). Another study assessed the ability of aspirin to protect mice exposed to a mixed field of neutrons and photons and a single or fractionated simulated GCR exposure (Holden et al., 2021). While data suggested that radiation-induced changes in systolic cardiac function and mitochondrial respiration were mitigated, aspirin did not benefit in preserving behavioral or cognitive performance. A significant risk associated with regular aspirin intake is GI bleeding; however, there is a lower risk of GI bleeding-associated morbidity and mortality in individuals younger than 65 years (Patrignani and Patrono, 2016), which correlates to the NASA astronaut age range.

#### 6.2.2. Celecoxib

Another easily accessible anti-inflammatory, celecoxib (a COX-2 inhibitor), has also shown success in mitigating lung injuries following irradiation (Hunter et al., 2013). The drug reduced lung toxicity when given to irradiated mice twice daily over 40 days. In addition, COX-2 inhibitors are being studied in humans undergoing thoracic irradiation (Gore, 2004). Because this drug is known to be safe in humans in a long-term dosing scenario, along with its use to relieve joint injuries, it represents a product that could be useful to protect astronauts from space radiation.

#### 6.2.3. CDDO-Me

Some NSAIDs can affect tumors and the tumor microenvironment by blocking cell proliferation and promoting apoptosis but have been implicated in adverse cardiovascular events (Varga et al., 2017). Other anti-inflammatory agents, such as CDDO-Me (bardoxolone methyl), act on the Nrf2 pathway, thereby upregulating an antioxidant response. CDDO-Me has demonstrated positive results in reducing tumor induction when tested in space radiation-simulated environments (Eskiocak et al., 2010), and protecting normal breast and lung cells from cesium irradiation (El-Ashmawy et al., 2014). The CDDO-ME compound was tested in several clinical trials, primarily for kidney (NCT03918447) and lung (NCT02036970) diseases, and also recently in COVID-19 patients (NCT04494646). The drug (an oral capsule) was safe and well-tolerated in these clinical studies. These agents, while promising, present challenges with the possibility of exacerbating cardiovascular events similar to over-the-counter NSAIDs and should be evaluated carefully for unintentional side effects. More information on this molecule can be found elsewhere in this special issue (Fornace & Shay).

### 6.3. Anti-apoptotics

A direct consequence of radiation damage is cellular apoptosis, also called programmed cell death or interphase death since cells usually die during interphase within a few hours of irradiation. The loss of normal nuclear structure and DNA degradation characterizes apoptosis, which occurs among various cell types at higher radiation doses (Little, 2003); however, it can be induced by relatively low doses of radiation in some cells (Xin et al., 2014). Products that modulate cellular apoptotic pathways have been tested with NIAID (and in some cases, NCI and NASA) support, demonstrating preclinical efficacy for ARS. These include a modified monoclonal anti-ceramide antibody approach (CX-01) (Rotolo et al., 2012), a GS-nitroxide (JP4–039) (Goff et al., 2011), and a pre-implantation factor therapy (sPIF) (Shainer et al., 2016). Although these drugs were tested in high-dose irradiation animal models, and their safety in a long-term use scenario is unknown, they represent novel compounds that could impact outcomes of NASA interest after low dose irradiation.

### 6.4. Senotherapeutics

Senescence is a process in which cells lose their proliferative capacity but remain metabolically active. Senescent cells display specific molecular, biochemical, and morphological features, and cancer treatments such as radiation (Shao et al., 2014) and chemotherapy (Ewald et al., 2010) induce senescence in both normal and cancer cells. Because of an increased understanding of multiple roles in cancer, cellular senescence is a vital target in the fight against cancer (Prasanna et al., 2021). Similarly, the role of senescent cells in radiation-associated aging of hematopoietic stem cells has led to the study of senolytic drugs as potential MCMs for ARS (Chang et al., 2016). While senescence is an essential tumor-suppressive phenomenon, senescent cells share hallmark features with cancer cells, including developing resistance to therapy, evasion of immune surveillance, and genomic instability. Thus, shared vulnerabilities of senescent cells with cancer cells could be exploited to develop novel approaches to prevent and mitigate carcinogenesis. A summary of senotherapeutics, including drug classes and developmental stage, mechanisms of action, critical knowledge gaps, pitfalls, challenges, and opportunities in their development as anticancer agents is available (Prasanna et al., 2021), and NIH has identified several broad areas as a blueprint in senescence research (Roy et al., 2020). In the context of radiation, the effects of dose, dose rate, radiation type, and the response kinetics of cellular senescence have not been fully understood.

### 6.5. Statins

Statins are a class of drugs commonly used to treat hypercholesterolemia and atherosclerosis. They offer cross-risk mitigation due to their pleiotropic effects that target neuroprotection, antioxidant modulation, and effects on anti-inflammatory pathways. In studies where atorvastatin was delivered chronically, there was minimal radiation mitigation; however, when combined with an ACE inhibitor (ramipril), the two drugs mitigated radiation-induced disruption of neurogenic signaling (Jenrow et al., 2011). Other studies found that lovastatin could interfere with the development of lung complications after large doses of irradiation (Williams et al., 2004), and mitigate radiation-induced damage to the salivary gland in murine models (Xu et al., 2016). Simvastatin is undergoing clinical trial testing in patients receiving radiation therapy for brain metastases, as a means of neuroprotection (NCT02104193). Available human data on statins suggests a radioprotective mechanism in reducing GI toxicity (Wedlake et al., 2012); however, a Danish study looking at the risk of statin use reported an increase in ovarian mucinous tumors associated with their use (Baandrup et al., 2015). In addition, there is an established link between adverse side effects and statin use, which could interfere with the ability of NASA personnel to carry out flight tasks.^10^ Nevertheless, given the benefits and proven long-term use of statins, NASA could consider this MCM when planning studies to evaluate radioprotectors and mitigators.

### 6.6. ACE inhibitors

The ability of ACE inhibitors to serve as radiation mitigators has been well described in studies reaching back to the 1980s (Ward et al., 1988). These products, licensed as anti-hypertensives, are used extensively in humans, so their safety profile, even with chronic use, has been well-characterized (Sidorenkov and Navis, 2014). A variety of ACE inhibitors (e.g., lisinopril, captopril, enalapril, ramipril, etc.) have shown efficacy in mitigating high-dose radiation injuries to the lungs (Kma et al., 2012; Medhora et al., 2012), kidneys (Moulder et al., 2014; Moulder et al., 2011), and other organs (Kohl et al., 2007; Jenrow et al., 2010). These products could be beneficial in the space environment, even at low doses, to interfere with injury processes induced by irradiation in these tissues.

### 6.7. Antibiotics

Antibiotics have been tested in acute radiation exposure scenarios. They have demonstrated efficacy in attenuating ARS, with antimicrobials such as tetracyclines (Kim et al., 2009; Epperly et al., 2010) and fluoroquinolones (Kim et al., 2009) showing promise as radiation mitigators. Antibiotics such as minocycline are also in clinical trials as neuroprotective treatments for patients undergoing metastatic brain tumor radiotherapy (Afshari et al., 2020). Further, NASA has investigated use of minocycline as a prophylactic MCM due to its antibiotic, anti-apoptotic, anti-inflammatory, and immunomodulatory properties (Mehrotra et al., 2013; Zhang et al., 2014). A second-generation, semi-synthetic tetracycline that has been used clinically for decades, minocycline is considered a safe drug in humans (Garrido-Mesa et al., 2013). Work performed by the U.S. Department of Defense (DoD) showed that ciprofloxacin, a quinolone antibiotic, improved survival and diminished symptoms in irradiated mice. It also improved neutrophil counts and function (Booth et al., 2012). In addition, ciprofloxacin enhanced recovery from hemorrhagic radiation proctitis in radiotherapy patients (Sahakitrungruang et al., 2011). Therefore, ciprofloxacin, with its ability to comprehensively modulate inflammation, immune signaling, and wound healing, has potential to mitigate radiation injuries arising from space-based exposures. Although this MCM could offer cross-risk protection for radiation-induced effects and the immune system to mitigate infection, it has been reported to increase intracranial hypertension (Milanlioglu and Torlak, 2011), which would not be acceptable for the crew since this complication is a risk outlined in the Human Research Roadmap. NASA should explore discussions with Human Health and Countermeasure (HHC) experts regarding this issue and investigate doses associated with intracranial hypertension.

## 7. Regulatory considerations

### 7.1. Working with the FDA for product approval

Understanding regulatory compliance is essential to successful development of products to address radiation normal tissue injuries, whether from planned (e.g., radiotherapy), unplanned (e.g., during a public health emergency) or contingent (space flight) exposure. The FDA is the U.S. regulatory agency tasked with protecting public health and ensuring the safety, efficacy, and security of drugs, biologics, and medical devices. Working within these regulatory guidelines, NIAID and NCI support research to develop therapeutics for FDA approval, whereas NASA has other mechanisms to allow for approval of products for administration to space flight personnel. Despite this different process, there is information to be gained by NASA through understanding the standard, NCI approach for therapies and the NIAID use of the FDA Animal Rule (21 CFR 314.600–650 for drugs; 21 CFR 601.90–95 for biologics), which was heavily influenced by the traditional clinical approval pathways used by NCI, and covers MCMs for which “human efficacy studies are not ethical or feasible” (Food and Drug Administration, 2015). Please see the manuscript by Chang et al. elsewhere in this special issue for additional information about this unique regulatory pathway for MCM development.

### 7.2. NASA’s regulatory approach

Given NASA’s unique environment, it is not feasible to perform experiments representative of what astronauts will be exposed to during space travel, and epidemiology provides minimal information. The level of FDA approval required for the NASA crew is determined by the NASA Office of Chief Health and Medical Officer. If an MCM shows promise as a radioprotector or a mitigator, it goes through the NASA Transition to Operations Review Process (TORP) to obtain approval for administration to crew for the demonstrated indication.^11^ Similar to, and in many ways patterned on FDA procedures, the TORP is designed to assess effectiveness and operational readiness of human health, human performance, and medical research and technology products and deliverables. These reviews require 1) a detailed description of the deliverable, its intended use, and a description of how it addresses a NASA-identified critical risk, medical, health, performance issues, or application; 2) data demonstrating efficacy or utility; 3) data demonstrating operational validation; 4) an implementation plan of how it is to be used or applied, 5) an analysis of mission resources necessary for implementation; and 6) a summary of developmental processes and milestones achieved. Similar to FDA Animal Rule approval, the NASA pathway requires extensive evidence in animal models, clinical safety data in humans and takes cues from the development of other radio-therapeutics, and is also based on traditional clinical approval processes used for NCI approaches.

## 8. Conclusion

There are several terrestrial-based radiation injury prevention products, either in routine clinical use or undergoing testing through other U.S. government programs such as NCI and NIAID, that could be used to address the health needs of humans in space and vice versa. Fortunately, careful coordination between funding agencies and scientists in the field has enabled the advancement of many approaches that could be useful for both NIH and NASA needs. Looking to the future, commercial space tourism is already a reality for sub-orbital flights and low-earth orbits, with private astronaut missions to the International Space Station currently underway, and moon trips expected during the next few decades. Although exposures may be limited due to shorter flight times, people in the general population will still be exposed to space radiation during these flights. As there are diverse tissue-specific cancer risk rankings across sex and race, including sex-specific organ risks, all of these factors will need to be considered as more people take these flights (Cucinotta and Saganti, 2022). Continued collaboration between organizations tasked with developing approaches to address radiation-induced injuries will ensure that products with the greatest promise are studied across all agencies’ mission-specific requirements and that human health will be well-addressed by NASA for their future long-term space flights. Similarly, advances made by NASA and their funded investigators can help NIH in their efforts to address radiotherapy complications and protection of civilians from other unintended radiation exposures.

## Figures and Tables

**Table 1:** Candidate ground-based MCM approaches with potential NASA chronic exposure interest. Research on many drugs shown in the table has been supported by multiple U.S. government organizations, enabling limited funding to be used to leverage research results across radiation mission spaces.

Product name	Source	Mechanism of action	Status	Funding agency(ies)	Reference(s)

JP4–039	University of Pittsburgh	Anti-apoptotic	R&D	NIAID, NCI, NASA	(Goff et al., 2011)
sPIF^‡^	BioIncept	Anti-apoptotic	R&D	NIAID	(Shainer et al., 2016)
Genistein	Various	Anti-apoptotic	GRAS	NIAID	(Prasanna et al., 2015; Day et al., 2008; Citrin et al., 2017; Zakeri et al., 2019)
CX-01	Ceramedix Holding, LLC	Anti-apoptotic	R&D	NIAID	(Rotolo et al., 2012)
BIO300	Humanetics	Anti-apoptotic SOD-mimetic	R&D	NASA, NCI NIAID	(Zakeri et al., 2019; Singh and Seed, 2020)
BMX-001	BioMimetix, JV, LLC	Anti-apoptotic SOD-mimetic	R&D	NCI	(Zakeri et al., 2019)
GC4419	Galera Therapeutics	Anti-apoptotic SOD-mimetic	R&D	NCI, NASA	(Anderson et al., 2018)
AEOL-10,150	Aeolus Pharmaceuticals	Anti-apoptotic SOD-mimetic	R&D	NIAID, NCI	(Murigi et al., 2015; MacVittie et al., 2017; Sonis, 2001)
Fluoroquinolones	Generic	Antibiotic	Licensed	NIAID	(Kim et al., 2009)
Minocycline	Generic	Antibiotic	Licensed	NASA, NCI	(Mehrotra et al., 2013; Zhang et al., 2014)
Tetracyclines	Generic	Antibiotic	Licensed	NIAID	(Epperly et al., 2010)
Aspirin	Generic	Anti-inflammatory	Licensed	NASA	(Qiao et al., 2018; Mitrugno et al., 2017; Camara Planek et al., 2020)
CDDO-ME	Various	Anti-inflammatory	R&D	NASA NCI	(Eskiocak et al., 2010; El-Ashmawy et al., 2014)
Celecoxib	Generic	Anti-inflammatory	Licensed	NIAID	(Hunter et al., 2013)
Bowman-Birk Inhibitor	University of Pennsylvania	Anti-inflammatory	GRAS (soybean-derived)	NASA, NCI	(Shaghagi et al., 2021; Kennedy, 2021)
Curcumin	University of Rochester	Anti-oxidant	GRAS^†^	NCI, NIAID	(Amini et al., 2018; Jelveh et al., 2013)
Flaxseed (dietary-derived)	Various	Anti-oxidant	GRAS	NCI, NASA, NIAID	(Lee et al., 2009; Christofidou-Solomidou et al., 2011)
LGM2605 (synthetic flaxseed derivative)	LignaMed	Anti-oxidant	R&D	NASA, NIAID	(Lee et al., 2009; Christofidou-Solomidou et al., 2011)
Vitamins (A, C, E)	Various	Anti-oxidant	Nutraceutical	NASA, NCI, NIAID	(Yoo et al., 2014)
Ketones	T Delta S	Anti-oxidant	GRAS	NIAID, NCI	(Klement, 2018)
Enterade	University of Florida	Increased GI absorption	Nutraceutical	NASA, NIAID	(Yin et al., 2014; Gupta et al., 2020)
ABT263	University of Arkansas for Medical Science	Senolytic	R&D	NCI, NIAID	(Chang et al., 2016)

* Research and Development.

† Generally Regarded as Safe (e.g., food additive).

‡ Preimplantation factor synthetic analog.

## Footnotes

1
https://www.nasa.gov/pdf/189195main_NASA_NIH_mou_20070912.pdf

2
https://www.nasa.gov/saa/domestic/27663_NASA-HHS_Interagency_Agreement_31July2018.pdf

3
http://go.nasa.gov/3DTMPSNRA

4
https://www.ncbi.nlm.nih.gov/books/NBK326791/

5
https://datascience.cancer.gov/data-commons

6
https://www.cancer.gov/about-nci/organization/ccg/research/structural-genomics/tcga

7
https://candle.cels.anl.gov/

8
https://www.immport.org/shared/home

9
https://federallabs.org/news/ketone-bodies-to-protect-tissues-from-damage-by-ionizing-radiation

10
https://www.acc.org/latest-in-cardiology/articles/2018/04/09/13/25/assessing-severity-of-statin-side-effects

11
https://nodis3.gsfc.nasa.gov/displayDir.cfm?Internal_ID=N_PR_8900_001B_&page_name=AppendixD
